# Depolymerization of Fucosylated Chondroitin Sulfate with a Modified Fenton-System and Anticoagulant Activity of the Resulting Fragments

**DOI:** 10.3390/md14090170

**Published:** 2016-09-21

**Authors:** Jun-hui Li, Shan Li, Zi-jian Zhi, Lu-feng Yan, Xing-qian Ye, Tian Ding, Lei Yan, Robert John Linhardt, Shi-guo Chen

**Affiliations:** 1 Zhejiang Key Laboratory for Agro-Food Processing, Department of Food Science and Nutrition, Fuli Institute of Food Science, Zhejiang University, Hangzhou 310058, China; 18511581374@163.com (J.L.); 11613001@zju.edu.cn (S.L.); zhizijian8866@163.com (Z.Z.); 11513026@zju.edu.cn (L.Y.); psu@zju.edu.cn (X.Y.); tding@zju.edu.cn (T.D.); 2 Center for Biotechnology & Interdisciplinary Studies, Department of Chemistry & Chemical Biology, Rensselaer Polytechnic Institute, Biotechnology Center 4005, Troy, NY 12180, USA; yuy8@rpi.edu (L.Y.); linhar@rpi.edu (R.J.L.)

**Keywords:** sea cucumber, fucosylated chondroitin sulfate, free-radical depolymerization, anticoagulant activity

## Abstract

Fucosylated chondroitin sulfate (fCS) from sea cucumber *Isostichopus badionotus* (fCS-*Ib*) with a chondroitin sulfate type E (CSE) backbone and 2,4-*O*-sulfo fucose branches has shown excellent anticoagulant activity although has also show severe adverse effects. Depolymerization represents an effective method to diminish this polysaccharide’s side effects. The present study reports a modified controlled Fenton system for degradation of fCS-*Ib* and the anticoagulant activity of the resulting fragments. Monosaccharides and nuclear magnetic resonance (NMR) analysis of the resulting fragments indicate that no significant chemical changes in the backbone of fCS-*Ib* and no loss of sulfate groups take place during depolymerization. A reduction in the molecular weight of fCS-*Ib* should result in a dramatic decrease in prolonging activated partial thromboplastin time and thrombin time. A decrease in the inhibition of thrombin (FIIa) by antithromin III (AT III) and heparin cofactor II (HCII), and the slight decrease of the inhibition of factor X activity, results in a significant increase of anti-factor Xa (FXa)/anti-FIIa activity ratio. The modified free-radical depolymerization method enables preparation of glycosaminoglycan (GAG) oligosaccharides suitable for investigation of clinical anticoagulant application.

## 1. Introduction

Fucosylated chondroitin sulfate (fCS) from sea cucumber has attracted increasing attention due to its potential therapeutic application, such as anti-human immunodeficiency virus (anti-HIV) activity [1], attenuation of renal fibrosis through a P-selectin-mediated mechanism [2], inhibition of tumor metastasis [3], and anti-hyperlipidemia activity [4]. The anticoagulant and antithrombotic properties make fCS a potential substitute for heparin [5,6]. The fCS polysaccharide has a different anticoagulant activity mechanism than heparin [7], and also causes undesirable side effects, including the activation of factor XII, platelet aggregation [8], hypertension and spontaneous bleeding in humans and some animals [7,8].

Depolymerization has been reported to be an effective way to decrease the adverse effects associated with fCS [9,10]. Depolymerized fCS exhibits high antithrombotic activity with reduced adverse effects, similar to those of unfractioned heparin (UFH) and low-molecular-weight heparin (LMWH) in rats and dogs [8,10]. The small scale degradation of fCS using a combination of hydrazine and nitrous acid [11] and ^60^Co irradiation [12] are effective for backbone depolymerization without loss of the fucose branches, which are a key functional group required for anticoagulation [13]. However, these methods are difficult to scale-up because they use toxic chemicals or radioactive ^60^Co. Acid-catalyzed hydrolysis and free-radical depolymerization are efficient ways for the large-scale preparation of low molecular fCSs [14]. However, acid-catalyzed hydrolysis can easily result in partial loss of sulfated fucose branches and their desulfation, significantly impacting the anticoagulant activity of the depolymerized fucosylated chondroitin sulfate products [13,15].

Free radicals generated using a Fenton system have been widely used to obtain low molecular weight (LMW) heparin or LMW dermatan sulfate having well defined compositions [15]. The extent of the free radical depolymerization can be controlled for the production of LMW heparin with excellent reproducibility [16]. Copper (II)-catalyzed Fenton system has been applied for the depolymerization of fCS using a H_2_O_2_ solution added using a peristaltic pump [17]. However, the preparation procedures are often complicated and uncontrollable. It is often difficult to control the titration rate of hydrogen peroxide, reaction time, pH shifts during the reaction, and the method results with a low yield of LMW fCS product. Furthermore, due to the poor reproducibility of these methods, it was difficult to efficiently control the degree of degradation, leading to possible problems with quality control during the production process. Therefore, there remains a need to develop efficient, controllable, economical and safe methods for preparing low molecular weight fCSs.

The fCS polysaccharide is a unique fucosylated chondroitin sulfate with a chondroitin sulfate type E (CSE) backbone, →4)-β-d-glucuronic acid (1→3)-β-d-4,6-*O*-sulfo-*N*-acetyl galactosamine (1→ and 2,4-di-sulfo fucose branches. First isolated from the sea cucumber, *Isostichopus badionotus* [18,19], fCS shows an activated partial thromboplastin time (APTT) of 183 IU/mg. This fCS also shows substantial antithrombin III (AT III) and heparin cofactor II HCII-mediated inhibition thrombin (FIIa) and factor Xa (FXa) activation. Furthermore, this fCS induced activation of factor XII (FXII) and caused bleeding in the animal studies, which limited its further use as an anticoagulant and antithrombotic drug. In the present study, we modified and optimized the Fenton reaction by the maintenance of pH with buffer and through the complete addition of H_2_O_2_ at the initiation of the reaction, with aim of establishing a controlled depolymerization to repeatably obtain a mixture of oligosaccharide fragments of different molecular weights. The mechanism of the depolymerization reaction was investigated using polyacrylamide gel electrophoresis (PAGE), gel permeation chromatography (GPC) and nuclear magnetic resonance (NMR) spectroscopy. The anticoagulant activities of the resulting fCS fragments were evaluated, using APTT, and thrombin time (TT), prothrombin time (PT), inhibition of FIIa by HCII and AT FXa by AT, with aims to investigate the anticoagulant mechanism.

## 2. Results and Discussion

### 2.1. Effect of Reaction Conditions on the Molecular Weights of Oxidative Depolymerized Products

**Figure 1:**
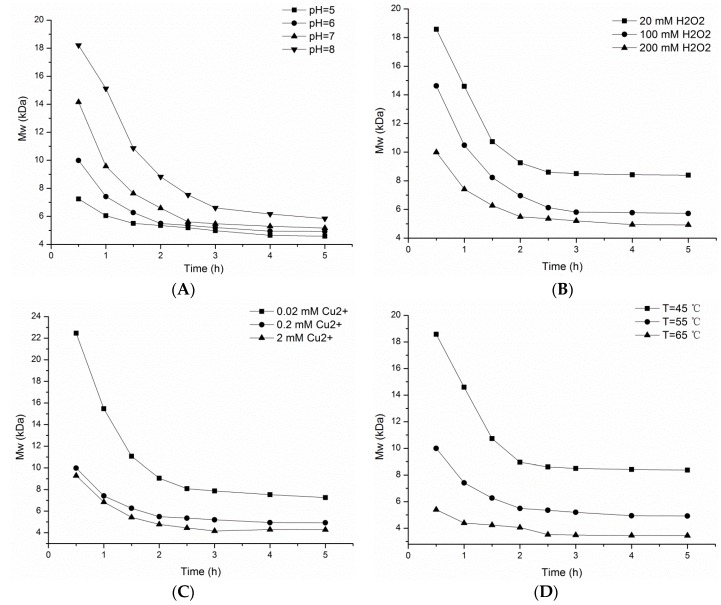
Effect of different reaction conditions on the molecular weights (Mws) of depolymerized fCS from sea cucumber *Isostichopus badionotus*. (**A**) pH; (**B**) the concentration of H_2_O_2_; (**C**) the concentration of Cu^2+^; and (**D**) reaction temperature.

The influence of pH, concentrations of H_2_O_2_ and Cu^2+^, reaction temperature, and reaction time were investigated to optimize depolymerization conditions using the Fenton-system. The pH of solution can affect the generation rate of the free radicals, altering the rate of polysaccharide depolymerization. A pH value of around 5 to 8 was applied in the optimization of depolymerization reaction to prevent the acidic or basic hydrolysis of the polysaccharides (Figure 1A). Polysaccharide molecular weight was sharply reduced in the first 2 h and then more slowly reduced over the next 3 h at all pH values examined. After 2 h of reaction, the amount of free radicals generated from H_2_O_2_ decreased due to the decomposition of hydrogen peroxide. Decreasing pH resulted in a reduced molecular weight for the depolymeirized polysaccharide products, consistent with previous reports that lower pH promoted faster depolymerization [20]. The amount of free radical generated depends on the concentration of H_2_O_2_ in the solution, which also affects the degradation rates (Figure 1B). Higher concentrations of H_2_O_2_ can produce more free radicals, which results in a significant difference in the molecular weight of the depolymerized products (Figure 1B). After 5 h of treatment, the molecular weights of depolymerized fCS from sea cucumber *Isostichopus badionotus* using initial H_2_O_2_ concentrations of 20 mM, 100 mM, 200 mM were approximately 9.0 kDa, 5.8 kDa and 4.2 kDa, respectively.

Copper is typically used as a catalyst in the depolymerization polysaccharide reaction and has been reported to achieve cleavage selectivity [21]. Increasing amounts of Cu^2+^ in the Fenton system can accelerate the depolymerization of fCS-*Ib*, resulting in depolymerized products molecular weights of 4.3 kDa, 6.2 kDa and 7.2 kDa, respectively, after 5 h of reaction (Figure 1C). High amounts of Cu^2+^ significantly improved the depolymerization within concentrations study. A concentration of 0.2 mM Cu^2+^ was chosen for our reaction conditions to perform catalyst under milder chemical condition and make the degradation highly controllable and reproducible [17]. Higher temperature results higher average kinetic energy and more molecular collisions per unit time [22]. Therefore, higher reaction temperatures can result in a faster polysaccharide depolymerization rate (Figure 1D). After 5 h of reaction, the molecular weight of the depolymerized products were 8.3 kDa, 5.7 kDa and 3.5 kDa, at 45 °C, 55 °C and 65 °C, respectively. Since high temperatures can destroy the sugar unit and decompose the H_2_O_2_ [23], 55 °C was chosen as the optimal reaction temperature.

Based on results obtained, we set the optimum values of pH 6.0, 200 mmol/L H_2_O_2_, 0.2 mmol/L Cu^2+^ and 55 °C as our reaction conditions for the rapid and controllable depolymerization of polysaccharide.

### 2.2. Free Radical Degradation of fCS-Ib in a Controllable Fenton System

The structure of the degradation products was investigated to better understand the structural changes that take place to fCS-*Ib* during the Fenton-reaction. Samples were treated under these optimized conditions for 1 h, 3 h and 5 h and their structure and anticoagulant activity were analyzed. The products were named DfCS-1, DfCS-3 and DfCS-5, respectively.

#### 2.2.1. GPC, PAGE and Chemical Compositional Analysis

Molecular weight analysis by GPC (Table 1) suggested the fCSs were depolymerized to 7.4 kDa, 5.2 kDa and 4.3 kDa after 1 h, 3 h and 5 h of degradation (DfCS-1, DfCS-3 and DfCS-5). Chemical compositional analysis indicated after the oxidation, the monosaccharide composition of the fCS-*Ib* remained unchanged (Table 2), suggesting that backbone chain of depolymeirized products still kept a typical chondroitin sulfate structure and oxidative depolymerization of fCS resulted no obvious loss of fucose branches, the key factor for the anticoagulant and antithrombotic activity of the fCSs. However, a slightly decreased amount in glucuronic acid (GlcA) content was observed, indicating that chain breakage might happen at this site.

**Figure 2:**
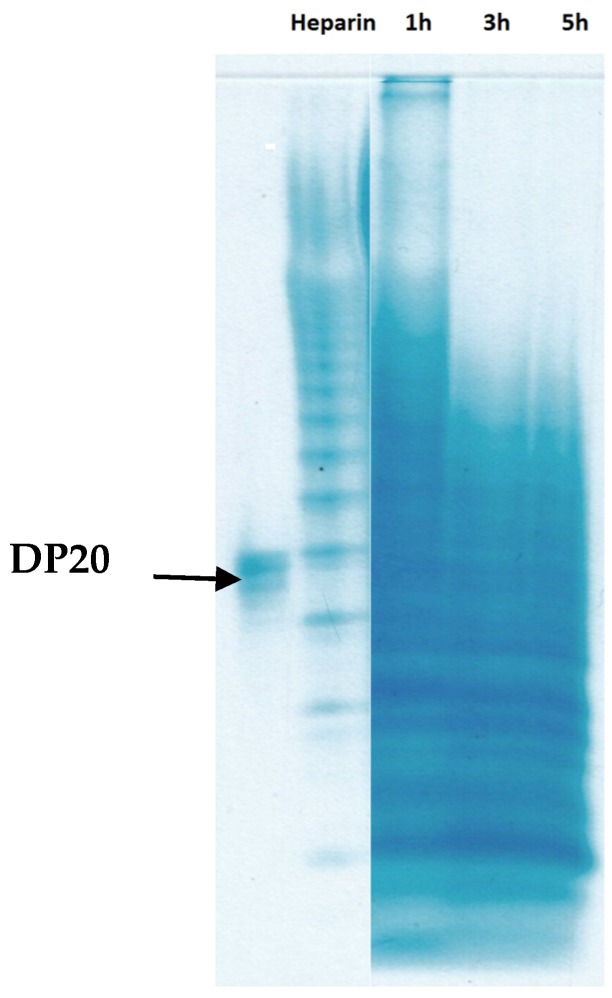
Polyacrylamide gel electrophoretograms of the fCS-*Ib* hydrolytic products. The products formed in the course of oxidative degradation with Fenton system were analyzed at different intervals with a 22% gel.

Further PAGE analysis, which exhibited a series of sharp bands (Figure 2), suggested that with the proceeding of hydrolysis from 1 to 5 h, the proportions of bands with higher electrophoretic mobility increased, and those clear bonds that appeared also suggested selective degradation of the reaction.

#### 2.2.2. NMR Analysis of the Degradation Products

**Figure 3:**
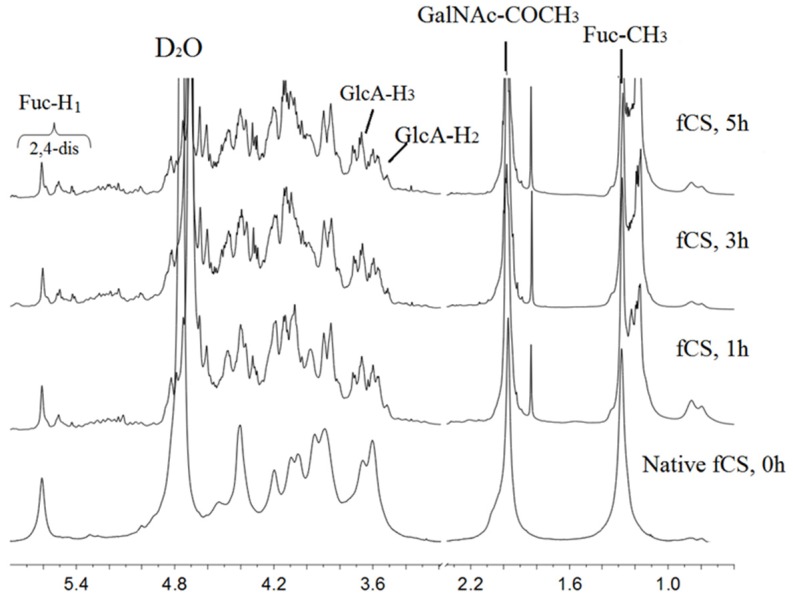
^1^H nuclear magnetic resonance (NMR) spectra (800 MHz at room temperature) of the native and three depolymerized fCS-*Ib* samples. The assignment of the peak is explained in the figure and the references [18].

The ^1^H NMR spectra of depolymerized products (Figure 3) were obtained to investigate the structural changes of fCS-*Ib* during oxidation. The results showed that the basic structure of the polysaccharide was nearly unchanged after depolymerization. The signals at 1.8–2.1 ppm and 1.1–1.4 ppm can be easily assigned to the methylprotons (CH_3_) of *N*-acetyl-d-galactosamine (GalNAc) and fucose (Fuc), respectively, and those signals among 3.0–4.8 ppm were attributable to the cross-ring protons. The chemical shift did not change compared with the native fCS-*Ib*, so it can be concluded that oxidative degradation does not impact the Fuc and GalNAc residues. In the anomeric region, the signal of the chemical shifts at ~5.61 ppm were assigned to the 2,4-*O*-di-sulfo fucose branches, and the typical anomeric proton signals of various sulfated fucose residues agreed with our previously published values [19].

However, there were some obvious changes following depolymerization. In the anomeric region, new signals appeared around 5.51 ppm that could be assigned to those fucose residues affected by the oxidation process, and the increase of this signal indicated that more severe reactions had occurred. Signals around 3.58–3.7 ppm assigned to H-2 and H-3 of glucuronic acid showed a substantial decrease as a function of degradation time and new signals at 3.5–3.68 ppm increased, which may be attributed to reduced chain size and degradation of GlcA at the reducing terminus. These results are also indicative of chain scission by free radicals generated by the Fenton system through their action on the glucuronic acid residues. The reduction of terminal GlcA also affected the other nearby signals, which induced the multi-distribution of the signals around 1.2–1.4 for fucose CH_3_ and 1.8–2.0 for GlcNAc-COCH_3_.

Thus, from ^1^H NMR, we conclude that the free radicals from the optimized Fenton system selectively acted on the GlcA, which is supported by a previous report that GlcA residues of glycosaminoglycans are very susceptible to free radical degradation [24]. These results are different from those previously obtained using ^60^Co irradiation [12]; here, the free radicals showed no selectivity and generated no clear changes in the signals of GlcA H-2 and H-3.

**Figure 4:**
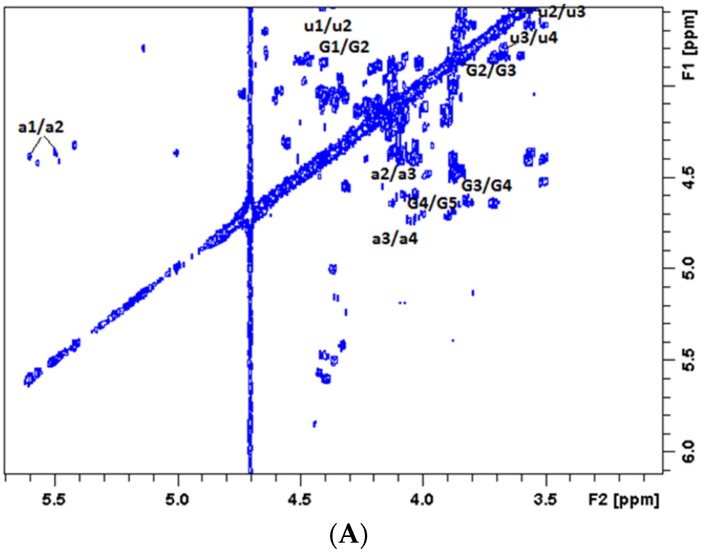

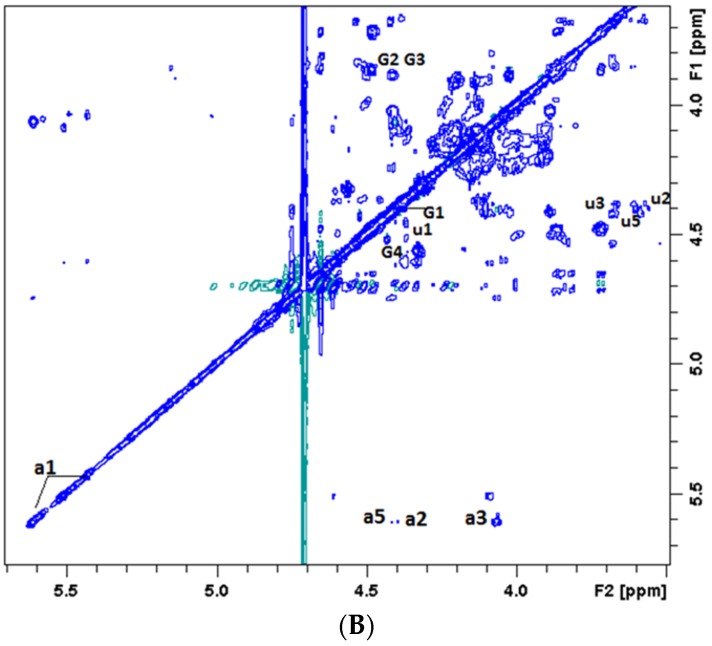
The 2D NMR spectra of DfCS-5 prepared by Fenton system (pH 6.0) at the concentration of 0.2 mol/L H_2_O_2_ and 0.2 mmol/L Cu^2+^ and at 55 °C: (**A**) Correlation spectroscopy (COSY) and (**B**) Total correlation spectroscopy (TOCSY). Signals designated with a reference to those produced by Fuc2,4S; and signals designated with G and u refer to *N*-acetyl-d-galactosamine (GalNAc) and glucuronic acid (GlcA), respectively.

The detailed assignment of the signals was further confirmed from the 2D NMR of the depolymerization products, prepared following a 5 h treatment (DfCS-5). Assignment of ^1^H and ^13^C chemical shifts of fucose branches and the CSE backbone in DfCS-5 were made from correlation spectroscopy (COSY) (Figure 4A), total correlation spectroscopy (TOCSY) (Figure 4B), heteronuclear single quantum coherence (HSQC) (Supplementary Figure S1A) and nuclear overhauser effect spectroscopy (NOESY) (Supplementary Figure S1B) spectra. The results confirmed that the signals at 5.51–5.45 could be assigned to 2,4-*O*-di-sulfo fucose branches, which might be associated with fucose at the terminus of the depolymerized chain. By combining COSY, TOCSY with HSQC, the new signals at 3.51, 3.67 ppm in ^1^H and 75, 72.9 ppm in ^13^C were assigned to the H-2 and H3 and C2 and C3 to GlcA (Supplementary Table S1), respectively. These signals were shifted to downfield, compared to those of native fCS-*Ib*.

### 2.3. In Vitro Anticoagulant Activity Analysis of Oxidative Degradation Products

Native fCSs have side effects such as the activation of factor XII and prolonged bleeding. Thus, by decreasing chain size using various methods, these adverse effects might be reduced or eliminated [10]. In the present study, the anticoagulant properties of depolymerized fCS-*Ib*, prepared using the optimized Fenton system, were analyzed using APTT, TT, PT and inhibition assays of thrombin (FIIa) and factor Xa (FXa) by antithrombin III (AT III) and heparin cofactor II (HCII).

The APTT assay determines interference with the intrinsic coagulation cascade and TT examines the last step of the coagulation cascade, thrombin-mediated fibrin formation [25]. The effects of native fCS and its depolymerized products on anticoagulant activities are summarized in Table 3. Native fCS-*Ib* showed an APTT of 183 IU/mg, a little higher than the standard heparin (212 IU/mg) used. Reduction in molecular weight by Fenton reaction for 1, 3 and 5 h, reduced the APPT values to 103.8 IU/mg, 60.5 IU/mg and 34.8 IU/mg, respectively. However, the TT-prolonging activities of depolymerized products were more significantly diminished than the APTT, as these were below 1 IU/mg for DfCS-3 and DfCS-5. None of the depolymerized products showed an observable effect on PT. Therefore, in contrast to the native polysaccharides that can act on both the intrinsic and extrinsic pathway, the low molecular weight derivatives exhibit anticoagulant activity only by inhibiting extrinsic coagulation. The variation between native polysaccharides and its depolymerized products on APTT and TT clearly suggest a different anticoagulant mechanism.

**Figure 5:**
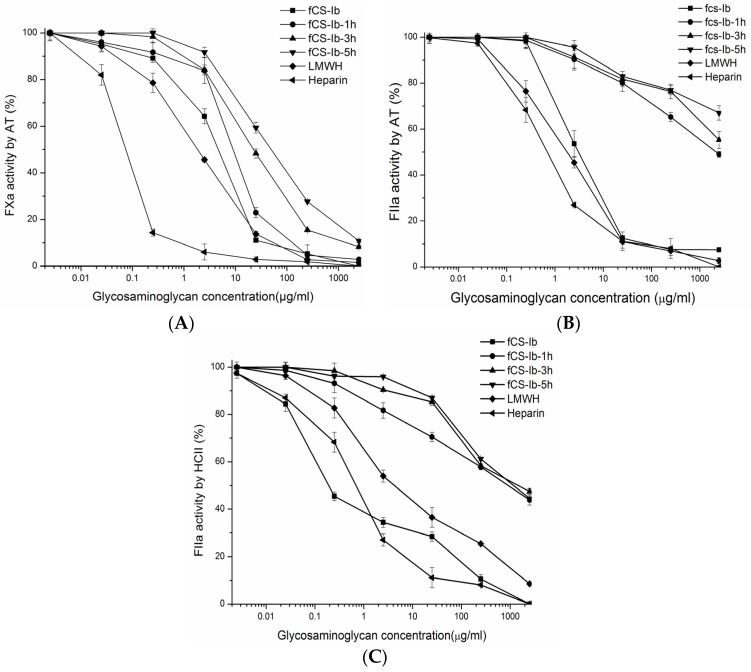
Effect of fCS-*Ib* and its depolymerized products on inhibition of FIIa and FXa activity. (**A**) FXa/AT; (**B**) FIIa/AT; (**C**) FXa/HCII. AT (1 IU/mL) or HCII (0.5 mmol/L) were incubated with FIIa (20 IU/mL) or FXa (0.4 IU/mL) in the presence of fCS and its depolymerized products at various concentrations. After 120 s of incubation at 37 °C, the remaining FIIa or FXa was determined with a chromogenic substrate (A405 nm/min). Results were shown as means ± SD (*n* = 3/group).

Based on the results of the coagulation-based assays, the inhibition of FIIa and FXa by AT and HCII and the anti-Xa/anti-IIa ratio, using defined amidolytic assays, were investigated and compared with unfractionated heparin LMWH (Figure 5) to help clarify anticoagulant properties and mechanism of action. The results (Figure 5A) indicated that both native fCS-*Ib* and its depolymerized products enhance inactivation of FXa by AT, and the enhancement decreased lightly with the reduction of molecular size. Higher concentrations resulted in greater inhibition of FXa by AT. Both the fCS-*Ib* and DfCSs can nearly complete inhibition of FXa through AT and was achieved at the concentration of 2500 μg/mL. The concentrations for half maximum FXa (EC_50_) were 4.7 μg/mL, 8.9 μg/mL, 22.8 μg/mL and 52.9 μg/mL for native fCS-*Ib*, DfCS-1, DfCS-3 and DfCS-5 (Table 3), respectively. The inhibitory activity was still very high even after a 5 h depolymerization reaction.

The AT mediated anti-FIIa inhibition effect was also concentration-dependent (Figure 5B), but the inhibitory effect of all of depolymerized products was much weaker than native fCS-*Ib*. At a concentration of 2500 μg/mL, fCS-*Ib*, DfCS-1, DfCS-3 and DfCS-5 afford 100%, 50%, 44% and 33% inhibition of FIIa activation by AT, respectively. Compared with native fCS, the sharply reduced ability of depolymerized products to inactivate thrombin is likely due to their relatively lower binding affinity to AT and when the molecular weight of depolymerized products is reduced to 7.3 kDa, the binding properties may be lost. These results are consistent with previous study by Wu et al., which showed that the intensity of AT-mediated anti-FIIa and anti-FXa activities of fCS and its depolymerized products decreased dramatically with decreasing molecular weight [7]. Furthermore, these results demonstrated that the anticoagulant properties of depolymerized holothurian glycosaminoglycan were quite different from those of depolymerized heparin in terms of antithrombin III-dependency. The decreased inhibitory effect of thrombin mediated by AT of depolymerized fCS may be related to its negligible risk of bleeding [8].

All of the depolymerized products showed no significant difference in the inhibition of FIIa activity by HCII and resulted in nearly 60% inhibition of thrombin activation by HCII at a dose of 2500 μg/mL, much lower than native fCS (100% inhibition) (Figure 5C). The anti-Xa/anti-IIa activity ratio of native polysaccharides was lower than heparin and LMWH, while the anti-Xa/anti-IIa activity ratio of depolymerized products was much higher than heparin (Table 3). Indeed, the anti-Xa activity of the depolymerized products was always much stronger than anti-IIa activity of the depolymerized products, leading to the increase of the anti-Xa/anti-IIa ratio (Table 3). These results were consistent with previous reports that anticoagulant and antithrombotic activity of native polysaccharides and its depolymerized products may be related to multiple-mechanisms and that they have different main targets [7,26]. We have discovered that oxidative depolymerization can significantly increase the anti-Xa/anti-IIa activity ratio of fCS and reduce anti-factor IIa activity relative to anti-factor Xa activity, which indicates that depolymerized fCS performs its major anticoagulant effect by activating AT, which mainly acts on FXa and, thus, should reduce side effects [8]. Unlike native fCS, the enhancement of anti-FXa/anti-FIIa activity ratio by depolymerization of heparin is much lower and the apparent differences between these polysaccharides further suggest differences in their anticoagulant mechanisms. In addition, the DfCS-1 showed higher APTT and lower inhibitory effect of thrombin and FXa by AT III and HCII than LMWH, indicating that there are other target enzymes for inhibition of the intrinsic coagulation pathway, and the anticoagulant mechanisms of depolymerized products were also different from LMWH.

## 3. Experimental Section

### 3.1. Isolation and Purification of fCS-Ib

Crude sea cucumber polysaccharides were prepared following the method reported previously [18]. Briefly, the sea cucumber body wall (~1 g) was dried, minced, and homogenized. The homogenate was treated with CHCl_3_/MeOH (4:1, *v*/*v*) to remove lipids before autoclaving at 50 °C for 4 h. The resulting residue was digested with 100 mg papain in 30 mL of 0.1 M sodium acetate buffer solution (pH 6.0) (5 mM EDTA and 5 mM cysteine) at 60 °C for 10 h. The digested mixture was centrifuged (4500× *g*, 10 min, 4 °C) and the polysaccharide in the clear supernatant was precipitated with 1.6 mL of 10% aqueous hexadecylpyridinium chloride solution. After standing at room temperature for 24 h, the mixture was centrifuged (4500× *g*, 10 min) and the precipitated polysaccharide was collected and re-dissolved in 10 mL of 3 M NaCl:ethanol (100:15, *v*/*v*) before further precipitation with 3 mL of 95% ethanol. After standing at 4 °C for 24 h, the precipitate formed was collected by centrifugation (2000× *g*, 15 min). The precipitate was dissolved in water and dialyzed against distilled water. The polysaccharide solution was lyophilized before analysis. The crude polysaccharide was further purified by anion-exchange chromatography on a diethylaminoethyl cellulose (DEAE–cellulose) column (2.6 cm × 40 cm) with elution by a linear gradient of NaCl, 0–1.2 M NaCl (in 0.1 M sodium acetate, pH 5.0) in 1000 min at a flow rate of 1.0 mL/min. Carbohydrate fractions were detected by phenol/sulfuric assay.

### 3.2. Free Radical Degradation of fCS-Ib in a Modified Fenton System

The depolymerized fCS-*Ib* fragments were prepared by modified free-radical depolymerization induced by Cu^2+^ catalyzed Fenton system [27]. Reaction conditions including pH (from pH 5.0 to pH 8.0), concentration of H_2_O_2_ (from 20 mM to 200 mM), Cu^2+^ (from 0.02 mM to 2 mM) and temperature (from 45 °C to 65 °C) were optimized. The fCS-*Ib* (200 mg) was dissolved in 100 mL 0.1M sodium acetate-acetic acid solution containing copper (II) acetate and adjusted the valve of pH. Hydrogen peroxide was added with mixing and maintained certain temperature for 5 h. Chelex 100 resin was added to terminate the reaction by removing Cu^2+^. The depolymerized products were desalinated by dialysis with a 500 Da cut-off membrane for 72 h, concentrated and subsequently lyophilized.

The degradation degree was analyzed by polyacrylamide gel electrophoresis (22%) and by high performance gel permeation chromatography (GPC). The GPC was performed on a Waters Ultrahydrogel 250 column (3.9 × 300 mm) (Milford, MA, USA) eluted by 0.2 M NaCl aqueous solution at the flow rate 0.5 mL/min monitored with a refractive index detector. Glucan standards are used to determine the molecular weight of the samples.

### 3.3. Chemical Composition Analysis of Oligosaccharide Fragments

Monosaccharide composition of oligosaccharide fragments was determined by the 1-phenyl-3-methyl-5-pyrazolone high performance liquid chromatography (PMP-HPLC) method [12]. Briefly, approximately 2 mg of oligosaccharide fragments was hydrolyzed with 4 M trifluoroacetic acid (TFA) at 110 °C for 8 h. After cooling to room temperature, TFA was then removed and the reaction solution was adjusted to pH 7.0 with 2M NaOH, and then with 0.3 M NaOH. The hydrolysate was derivatized with 50 μL of 0.3 M NaOH and 50 μL of 0.5 M PMP solution at 70 °C for 100 min. Chloroform was used to extract the hydrolysate and the hydrolysate was analyzed by HPLC with an ZORBAX Eclipse XDB-C18 column (Agilent, 5 μm, 4.6 mm × 250 mm, Santa Clara, CA, USA). The mobile phase A was aqueous containing sodium phosphate buffer (0.05 M, pH 6.9) and acetonitrile (*v*/*v*; 85:15) and the mobile phase B was aqueous containing sodium phosphate buffer (0.05 M, pH 6.9) and acetonitrile (*v*/*v*; 60:40). The time program of HPLC analysis was 0→10→30 min and the concentration program was 0→8%→20% of the mobile phase B at a flow rate of 1 mL/min and the samples were detected by UV detection at 250 nm, and the injection volume was 20 μL.

### 3.4. NMR Analysis of Oligosaccharide Fragments

For NMR spectroscopic analysis, native polysaccharide or oligosaccharide mixtures (20 mg) were dissolved in 500 μL of D_2_O (99.8%) and lyophilized three times to substitute the exchangeable protons, and finally dissolution in 500 μL of high quality D_2_O (99.96%) containing 0.1 μL acetone and then transfer to NMR microtubes. In addition, 1 H nuclear magnetic resonance (NMR) and homonuclear 1H/1H correlation experiments (COSY, TOCSY), nuclear Overhauser effect spectroscopy (NOESY), and heteronuclear single quantum coherence (HSQC) experiments were performed on a Hudson–Bruker SB 800 MHz Spectrometer (Madison, WI, USA) at room temperature.

### 3.5. Anticoagulant Assays

The activated partial thromboplastin time (APTT) and thrombin time (TT) assays were determined with a coagulometer (RAC-120, China) using APTT and TT reagents and standard human plasma as previously described [1]. The results were expressed as international units/mg using a parallel standard curve based on the International Heparin Standard (212 IU/mg).

### 3.6. Inhibition of Thrombin or FXa by AT III and HCII in the Presence of fCS-Ib and Its Depolymerized Products

The inhibition experiments were carried out in a 96-well micro-titerplate as described [9,19]. The reactant solutions included AT (0.5 IU/mL) or HCII (0.5 μmol/L) and samples or the standard heparin at different concentrations in 40 μL of Tris/polyethylene glycol (PEG) buffer (0.02 M Tris/HCl, 0.15 M NaCl and 1.0 mg/mL PEG 8000, pH 7.4). FIIa (40 μL of 5 IU/mL) or FXa (40 μL of 0.4 IU/mL) was added to initiate the reaction. After incubation at 37 °C for 60 s, 40 μL of TS/PEG buffer containing 0.625 mM colorimetric substrate of FIIa or 1 mM chromogenic substrate SXa-11 of FXa was added and the absorbance at 405 nm was measured at intervals of 15 s within a period of 300 s in a micro-plate reader [25]. The absorbance change rate was proportional to the FIIa and FXa activity remaining in the incubation mixtures. Heparin was used as a control and the experimental results were expressed as the percent of control (*n* = 3).

EC_50_ values were obtained by fitting the data to a noncompetitive inhibition model for the glycosaminoglycans according to Sheehan and Walke [28]. The anti-Xa/anti-IIa ratio was calculated using a standard curve of different concentrations of unfractionated heparin (0.1–2 IU/mL).

## 4. Conclusions

In the present study, a modified controllable Fenton-system was adopted to depolymerize the fucosylated chondroitin sulfate from sea cucumber, *I. badionotus* (fCS-*Ib*). The depolymerization conditions were optimized and the results indicated that lower pH, higher concentration of hydrogen peroxide and reaction temperature and longer time can increase the depolymerization efficiency. Chemical composition, PAGE and NMR analysis indicated the composition of the polysaccharides was almost unchanged during depolymerization, whereas the free radicals preferentially cleaved the GlcA in the backbone, which were different from other phytochemical methods [29]. Anticoagulant assays of the degradation fragments indicated the reduction in molecular weight resulted in a decrease of APTT/TT-prolonging activity, but the anticoagulant activity remained high after a 5 h depolymerization. Further anticoagulation assays on the depolymerization products suggested their inhibitory effects of thrombin mediated through AT/HCII were sharply reduced after depolymerization, whereas the inactivation of FXa mediated by AT was only slightly affected, which indicated that the depolymerization products of the fCS-*Ib* may selectively act on the intrinsic pathway of coagulation. In addition, a sharp increase in anti-Xa/anti-IIa ratio of depolymerized products suggests that controlling molecular weight is critical in controlling the side effects of depolymerized fCS, although additional studies are required to clarify the mechanism in terms of action.

## Figures and Tables

**Table 1:** Molecular weight of native fucosylated chondroitin sulfate (fCS) from sea cucumber *Isostichopus badionotus* (fCS-*Ib*) and its depolymerized products, unfractionated heparin (UFH) and commercial low molecular weight heparin (LMWH).

Samples	Average of Molecular Weight (*n* = 3)		Polydispersity (Mw/Mn)
	Weight Average (Mw) (kDa)	Number-Average (Mn) (kDa)	
fCS*-Ib*	109 ± 6.13	94.78 ± 3.97	1.15 ± 0.04
DfCS-1	7.4 ± 0.486	4.60 ± 0.36	1.61 ± 0.02
DfCS-3	5.2 ± 0.140	2.89 ± 0.41	1.80 ± 0.18
DfCS-5	4.3 ± 0.126	2.38 ± 0.40	1.80 ± 0.21
LMWH	6.4 ± 0.538	5.0 ± 0.55	1.28 ± 0.03
UFH	18.6 ± 0.224	13.47 ± 0.58	1.38 ± 0.04

**Table 2:** Chemical composition of native fCS-*Ib* and its depolymerized products.

Samples	Mw (kDa)	Molar Ratios ^a^		
		GlcA	GalNAc	Fuc
fCS-*Ib*	109	1.43	1	1.71
DfCS-1	7.4	1.35	1	1.70
DfCS-3	5.2	1.32	1	1.72
DfCS-5	4.3	1.30	1	1.71

^a^ Molar ratio is expressed as relative to GalNAc. GlcA: Glucuronic acid; GalNAc: *N*-acetyl-d-galactosamine; Fuc: fucose.

**Table 3:** Anticoagulant properties of fCS-*Ib* and its depolymerized products

Samples	Mw (kDa)	APTT */TT * (IU/mg)	EC_50_ (μg/mL) (Anti-FIIa/AT) **	EC_50_ (μg/mL) (Anti-FIIa/HCII) **	EC_50_ (μg/mL) (Anti-FXa/AT) **	Anti-Xa/Anti-IIa
fCS-*Ib*	109	187 157	3.2	0.2	4.7	0.2
DfCS-1	7.4	103.8 34.3	> 1500	857.5	8.9	88
DfCS-3	5.2	60.5 < 1	> 1500	1490	22.8	42
D-fCS-5	4.3	34.8 < 1	> 1500	> 1500	52.9	38.6
Heparin	18.6	212 212	0.7	0.67	0.22	1
LMWH	6.4	69 64	1.82	5.45	2.35	4.1

* The activity is expressed as international units/mg using a parallel standard curve based on the International Heparin Standard (212 IU/mg); ** “Anti-FIIa/HCII” means effect of fCSs on inhibition of thrombin by HCII; “Anti-FIIa/AT” means inhibition of thrombin by AT; “Anti-FXa/AT” means inhibition of FXa by AT; Mw: Molecular weight; APTT: Activated partial thromboplastin time; TT: Thrombin time; FIIa: Thrombin; FXa: Activated factor X; AT: Antithrombin III; HCII: Heparin cofactor II; EC_50_: Concentration required to obtain 50% inhibition of activated anticoagulant factor.

## Supplementary Materials

The following are available online at www.mdpi.com/1660-3397/14/9/170/s1, Figure S1: The 2D NMR spectra of DfCS-5 prepared by Fenton-system (pH 6.0) at the concentration of 0.2 mol/L H_2_O_2_ and 0.2 mmol/L Cu^2+^ and at 55 °C: (A) Heteronuclear single quantum coherence (HSQC); (B) nuclear overhauser effect spectroscopy (NEOSY). Signals designated with a refer to those produced by Fuc2,4S; signals designated with G and u refer to *N*-acetyl-d-galactosamine (GalNAc) and glucuronic acid (GlcA), respectively, Table S1: Assignment of ^1^H/^13^C NMR signals of depolymerized products.
