# Supplementary material for: Depolymerization of Fucosylated Chondroitin Sulfate with a Modified Fenton-System and Anticoagulant Activity of the Resulting Fragments
Source: Mar Drugs. 2016 Sep 21;14(9):170. doi: 10.3390/md14090170 (PMC5039541; doi:10.3390/md14090170)
Supplement: Supplementary file 1 [file marinedrugs-14-00170-s001.pdf]

Supplementary Materials: Depolymerization of Fucosylated Chondroitin Sulfate with a Modified Fenton-System and Anticoagulant Activity of the Resulting Fragments

Jun-hui Li, Shan Li, Zi-jian Zhi, Lu-feng Yan, Xing-qian Ye, Tian Ding, Lei Yan, Robert John Linhardt and Shi-guo Chen

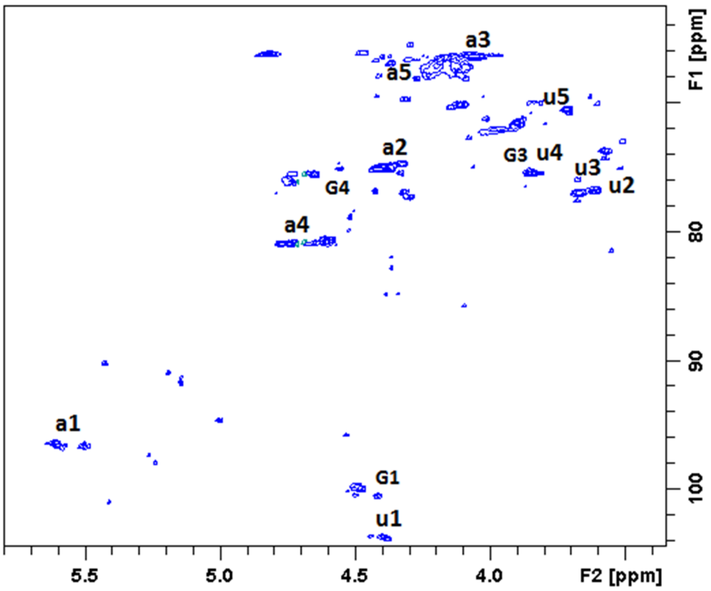

(**A**)

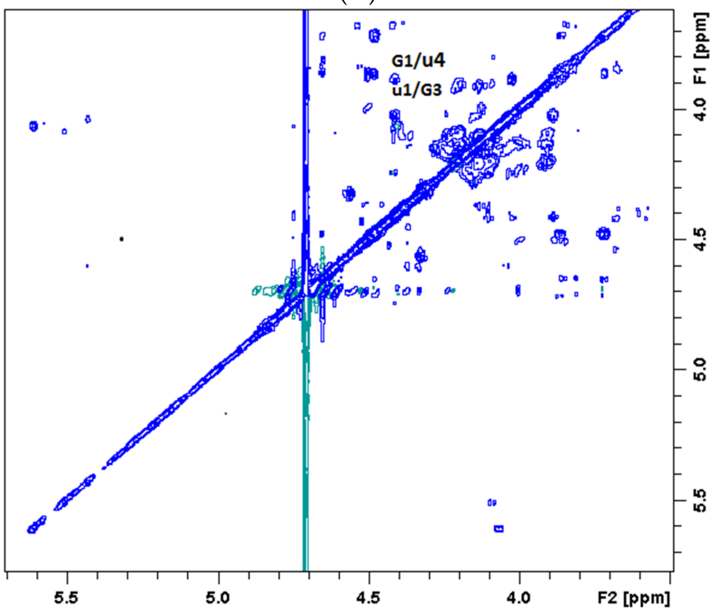

(**B**)

Figure S1. The 2D NMR spectra of DfCS-5 prepared by Fenton-system (pH 6.0) at the concentration of 0.2 mol/L H_2O_2 and 0.2 mmol/L Cu^{2+} and at 55 °C: (**A**) Heteronuclear single quantum coherence (HSQC); (B) nuclear overhauser effect spectroscopy (NEOSY). Signals designated with a refer to those produced by Fuc2,4S; signals designated with G and u refer to *N*-acetyl-D-galactosamine (GalNAc) and glucuronic acid (GlcA), respectively.

Table S1. Assignment of ^{1}H/^{13}C NMR signals of depolymerized products.

Samples	D-fCS-*Ib*-5h			Fuc0S **	CSE ***	
	GlcA (β)	GalNAc4,6S (β)	Fuc2,4S(α)	(Standard)	GlcA (β)	GalNAc4,6S (β)
H1 (C1)	4.39 (103.2)	4.4 (100.2)	5.60 or 5.5 (96.1)	5.03 -	4.49 (104.2)	4.65 (102.1)
H2 (C2)	3.51 (72.9)	3.85 (52.3)	4.39 (75.7)	3.96 -	3.41 (73.0)	4.07 (52.2)
H3 (C3)	3.67 (76.9)	3.87 (75.3)	4.09 (66.1)	4.01 -	3.60 (74.7)	4.07 (76.1)
H4 (C4)	3.80 (71.6)	4.59 (76.3)	4.73 (80.6)	3.96 -	3.80 (82.6)	4.80 (76.6)
H5 (C5)	-	-	4.42 (67.7)	4.35 -	3.68 (77.5)	4.10 (73.3)
H6 (C6)	-	-	-	1.21 -	-	4.30 (68.5)
CH3	-	1.92 (24.0)	-	-	-	-
C=O	- (175.2)	- (176.3)	-	-	-	-

** The chemical shifts of Fuc0S were from a standard non-sulfated monosaccharide fucose, cited from reference [1]; *** Chemical shifts of chondroitin sulfate type E (CSE) are cited from reference [1]; GlcA: Glucuronic acid; GalNAc: *N*-acetyl-D-galactosamine; Fuc: fucose.

References

1. Chen, S.G.; Li, G.Y.; Wu, N.; Guo, X.; Liao, N.B.; Ye, X.Q.; Liu, D.H.; Xue, C.H.; Chai, W.G. Sulfation pattern of the fucose branch is important for the anticoagulant and antithrombotic activities of fucosylated chondroitin sulfates. *Biochim. Biophys. Acta* **2013**, *1830*, 3054–3066.
